# AO Patient Outcomes Center: Design, Implementation, and Evaluation of a Software Application for the Collection of Patient-Reported Outcome Measures in Orthopedic Outpatient Clinics

**DOI:** 10.2196/10880

**Published:** 2019-04-12

**Authors:** Nan E Rothrock, Michael Bass, Andrea Blumenthal, Richard C Gershon, Beate Hanson, Alexander Joeris, Aaron Kaat, Suzanne Morrison, Robert V O'Toole, Shalini Patel, Michael Stover, Michael J Weaver, Raymond White, Maria Varela Diaz, Mark S Vrahas

**Affiliations:** 1 Department of Medical Social Sciences Feinberg School of Medicine Northwestern University Chicago, IL United States; 2 AO Clinical Investigation and Documentation AO Foundation Duebendorf Switzerland; 3 Department of Health Services University of Washington Seattle, WA United States; 4 Partners eCare Partners HealthCare Boston, MA United States; 5 Department of Orthopaedics University of Maryland School of Medicine Baltimore, MD United States; 6 Department of Orthopaedic Surgery Feinberg School of Medicine Northwestern University Chicago, IL United States; 7 Orthopaedic Surgery Maine Medical Center Portland, ME United States; 8 Department of Orthopaedics Cedars Sinai Health System Los Angeles, CA United States

**Keywords:** orthopedics, patient reported outcome measures, tablet computers

## Abstract

**Background:**

Patient-reported outcomes are increasingly utilized in routine orthopedic clinical care. Computer adaptive tests (CATs) from the Patient-Reported Outcomes Measurement Information System (PROMIS) offer a brief and precise assessment that is well suited for collection within busy clinical environments. However, software apps that support the administration and scoring of CATs, provide immediate access to patient-reported outcome (PRO) scores, and minimize clinician burden are not widely available.

**Objective:**

Our objective was to design, implement, and test the feasibility and usability of a Web-based system for collecting CATs in orthopedic clinics.

**Methods:**

AO Patient Outcomes Center (AOPOC) was subjected to 2 rounds of testing. Alpha testing was conducted in 3 orthopedic clinics to evaluate ease of use and feasibility of integration in clinics. Patients completed an assessment of PROMIS CATs and a usability survey. Clinicians participated in a brief semistructured interview. Beta-phase testing evaluated system performance through load testing and usability of the updated version of AOPOC. In both rounds of testing, user satisfaction, bugs, change requests, and performance of PROMIS CATs were captured.

**Results:**

Patient feedback supported the ease of use in completing an assessment in AOPOC. Across both phases of testing, clinicians rated AOPOC as easy to use but noted difficulties in integrating a Web-based software application within their clinics. PROMIS CATs performed well; the default assessment of 2 CATs was completed quickly (mean 9.5 items) with a satisfactory range of measurement.

**Conclusion:**

AOPOC was demonstrated to be an easy-to-learn and easy-to-use software application for patients and clinicians that can be integrated into orthopedic clinical care. The workflow disruption in integrating any type of PRO collection must be addressed if patients’ voices are to be better integrated in clinical care.

## Introduction

There is an increasing demand to utilize patient-reported outcomes (PROs) in clinical care for a variety of aims. PROs that measure symptoms (eg, depression) and disability (eg, physical function) can be utilized to monitor response to treatment, detect unrecognized problems, improve patient and provider communication, and possibly improve health outcomes [1,2]. PROs are particularly important within orthopedics as treatment is often initiated to improve a patient’s physical function and reduce pain. Quantification of the patient’s perspective can be utilized in treatment decision-making such as when the patient’s pain and disability have progressed enough to consider joint replacement and to help judge the success of treatment [3]. Payers have also introduced PROs as a method for assessing health care quality (PRO Performance Measures) rather than only utilizing measures that evaluate the process of care [4-6]. For example, the Centers for Medicaid and Medicare Services now include submission of PRO data for total hip and total knee replacement reimbursement as part of their Comprehensive Care for Joint Replacement Payment Model [7]. It is hoped that using PRO Performance Measures will alter the definition of health care quality to include the function and symptom burden of patients [8].

To improve measurement precision, efficiency, applicability, and interpretability, the National Institutes of Health invested in the development and validation of the Patient-Reported Outcomes Measurement Information System (PROMIS), a collection of PRO measures that assesses important domains of self-reported health. PROMIS measures include computer adaptive tests (CATs)—a tailored administration in which questions are selected dynamically on the basis of past responses using the most informative question for that respondent’s specific level of function or symptom severity. This offers rapid assessment with high measurement precision across a wide range of functional abilities and symptom severities. CATs are particularly suited for integration in clinical care as there is little time in the clinic workflow for assessment and a need for highly precise measures when evaluating individual patient data [9].

Our aim was to develop a software application that enables the administration and scoring of PROMIS CATs and other relevant PROs in the clinical routine across diverse orthopedic clinics. Specifically, we hypothesized that (1) we could develop a software application that is easy to learn and easy to use for clinicians and patients, (2) AO Patient Outcomes Center (AOPOC) is feasible for integration into orthopedic clinics, and (3) PROMIS CATs provide rapid and precise quantification of symptoms and function across a wide range of patients.

## Methods

### AO Patient Outcomes Center Design

#### Design Principles

AOPOC followed 5 design principles: (1) a primary focus on in-clinic data collection for use in the clinical encounter, including display of longitudinal PRO data for an individual patient, (2) a common assessment battery of PROMIS CATs for all patients, (3) the capability to add other patient- and clinician-reported measures to the assessment, (4) an easy-to-learn and easy-to-use interface that imposes minimal burden on surgeons, and (5) the ability to export data for a group of patients for research analyses.

#### Description of AO Patient Outcomes Center

**Figure 1:**
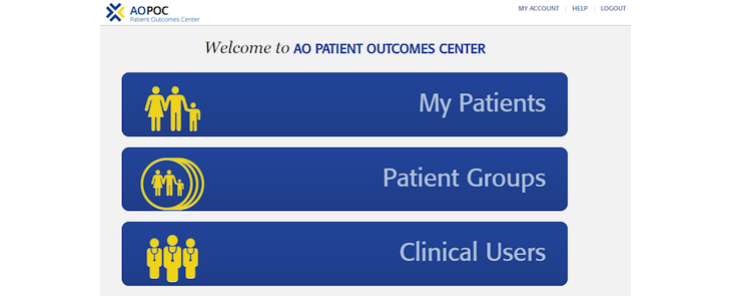
AO Patient Outcomes Center (AOPOC) homepage.

**Figure 2:**
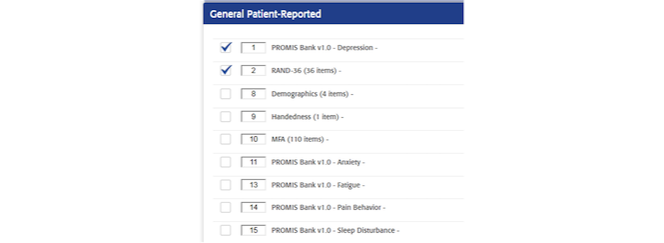
Portion of AO Patient Outcomes Center patient-reported outcomes library. MFA: Musculoskeletal Function Assessment; PROMIS: Patient-Reported Outcomes Measurement Information System.

**Figure 3:**
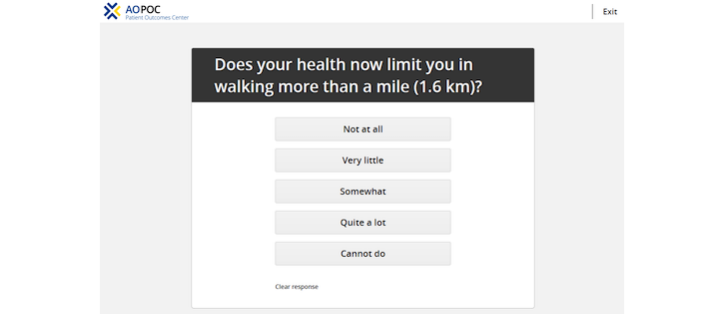
AO Patient Outcomes Center patient interface.

**Figure 4:**
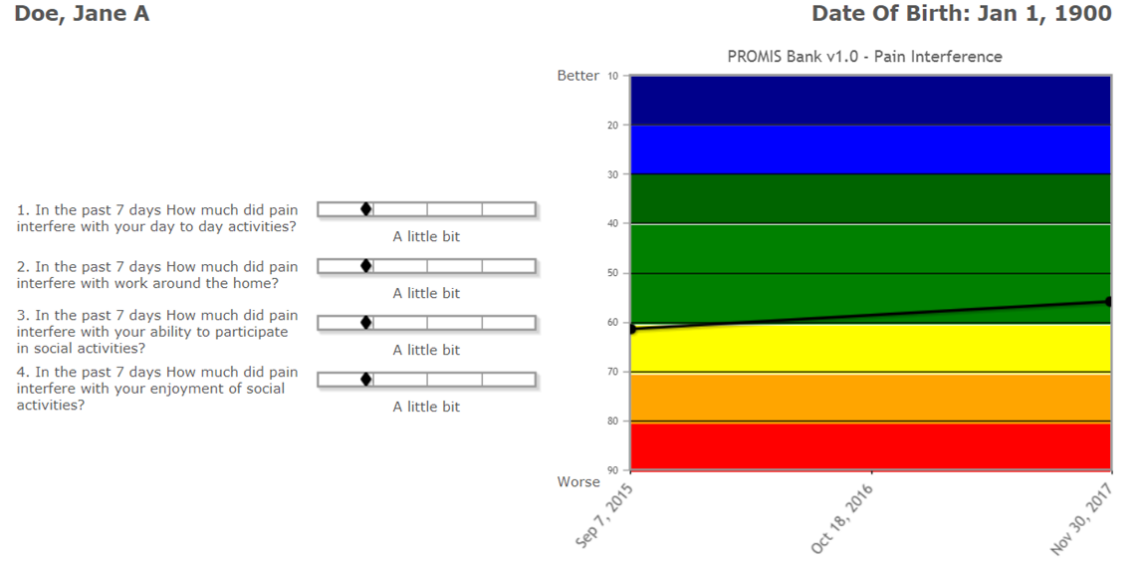
Portion of AO Patient Outcomes Center patient-reported outcomes report. PROMIS: Patient-Reported Outcomes Measurement Information System.

AOPOC is a Web-based software application to collect PROs in orthopedic clinics. A clinician is able to establish 1 or more *Patient Groups* (Figure 1). Assessment content can be tailored by the clinician for each Patient Group. For example, clinicians may utilize different PROs for individuals receiving total hip replacement versus an elbow injury. A library of high-quality PROs used in orthopedic care is available (Figure 2), as are numerous clinician-provided variables such as fracture classification code, mechanism of injury, and body mass index. All Patient Groups include the PROMIS CATs for Pain Interference and Physical Function. A patient is registered in AOPOC with full name, date of birth, and is assigned to 1 or more Patient Groups. The interface for the patient to complete the assessment is a simple design maximized for viewing on an iPad (Figure 3). Each CAT is scored in real time and displayed in a longitudinal graph along with the questions and responses from the most recent assessment (Figure 4). This report is available as a PDF to share with a patient or manually added to an electronic health record (EHR). Data from a Patient Group can be exported. They include raw response data, PROMIS measure scores, time and date of a patient’s responses, and response time. Optionally, multiple consent forms can be included to facilitate the use of AOPOC in research data collection.

A clinician can give access to a Patient Group to other clinical users who belong to the same organization. For example, a surgeon can establish a Trauma Patient Group and give access to fellows, front desk staff, and a physician assistant. Each member of the team has a unique log-in ID and password and is assigned a level of access to determine whether or not he or she can register patients, modify the assessment content, export data, or carry out other actions.

### Default Patient-Reported Outcome Battery

The default assessment includes 2 PROMIS CATs. A minimum of 4 and a maximum of 12 questions are administered per CAT. PROMIS T-scores have a mean of 50 (SD 10) in the US general population.

#### Patient-Reported Outcomes Measurement Information System v1.0 Pain Interference Computer Adaptive Test

Consequences of pain on the relevant aspects of one’s life are measured, including the extent to which pain hinders engagement with social, cognitive, emotional, physical, and recreational activities. Higher scores indicate more difficulties from pain.

#### Patient-Reported Outcomes Measurement Information System v1.2 Physical Function Computer Adaptive Test

Self-reported capability of physical activities including upper extremities, lower extremities, and central regions (neck, back), as well as instrumental activities of daily living, such as running errands are measured. The initial version of AOPOC utilized PROMIS CATs for Mobility (v1.2) and Upper Extremity Function (v1.2) instead of the single PROMIS Physical Function CAT.

### AO Patient Outcomes Center Development

To develop the application, we utilized a modified Agile methodology [10-12] that enabled iterative development with continuous feedback. A multidisciplinary team of orthopedic trauma surgeons, clinic staff, and PRO scientists compiled requirements and constructed use cases that included features’ functionality, terminology, navigation, and user interface. Software development, quality assurance (QA) testing, and user acceptance testing (UAT) were completed. When all test cases passed QA, AOPOC was made available in a Web-based production environment.

### Evaluation

**Figure 5:**
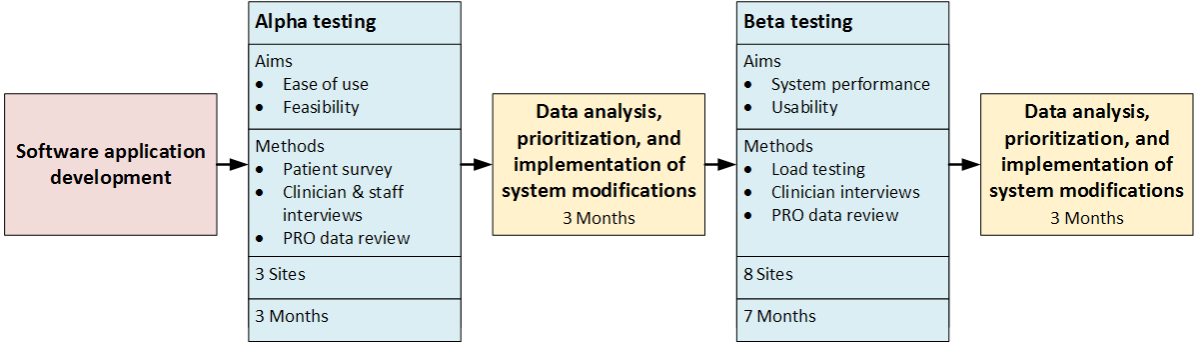
Stages of AO Patient Outcomes Center evaluation. PRO: Patient-Reported Outcomes.

AOPOC was evaluated in multiple waves of testing (see Figure 5). This included alpha testing for ease of use and feasibility and beta testing for system performance and usability.

### Alpha-Phase Testing

Alpha-phase testing was conducted to test ease of use and feasibility in integrating AOPOC in orthopedic clinics. Data were collected from both patients and clinic staff at 3 orthopedic trauma clinics in US academic medical centers associated with a surgeon in the project team. Each site’s Institutional Review Board determined that the project did not meet the criteria to be considered human subjects research.

#### Patients

The site lead identified the patient population for alpha testing (eg, single surgeon’s patients, specific clinic’s patients). Adult, English-speaking patients were eligible to participate. Participants were asked to complete a battery of PROs selected by his or her surgeon. Following the PROs, an 11-item usability survey comprising questions related to past computer use experience, comfort using the data collection device (eg, tablet computer), and satisfaction with the user interface was administered. Ease-of-use questions had 4 response options (0=Not at all, 1=A little bit, 2=Somewhat, 3=Quite a bit). User-interface questions utilized 5 response options (4=Excellent, 3=Very good, 2=Good, 1=Fair, 0=Poor).

#### Clinic Staff

After 3 to 9 weeks of AOPOC experience, the site lead identified staff including surgeons, other clinicians, and administrative personnel (eg, front desk staff) who interacted with AOPOC on more than 1 occasion. All were invited via email to participate in a 20-min semistructured interview by phone. The interview included open-ended questions targeting specific features of AOPOC including the following: (1) completing the application for implementing AOPOC at his or her site, (2) establishing the assessment content for specific patients (Patient Group set up), (3) enrolling clinical users, (4) registering patients, (5) having patients complete the assessment, and (6) accessing patient data. Multiple-choice questions assessing the ease of use of specific features, system usefulness, and degree of disruption to clinic workflow were also administered. Questions used a 5-point scale ranging from 1 (Not at all) to 5 (Very). The interview script was modular so that interviewees were only asked about those portions of AOPOC which they utilized. All issues, requested modifications, and responses to multiple-choice items were recorded in a database. In addition, communication with the AOPOC support desk was used to identify areas of confusion or errors as well as requested system modifications. This feedback was added to the database. Throughout alpha testing, bugs (instances when AOPOC was not functioning as it should) were distinguished from change requests (eg, modification to improve usability or expand system capabilities). Bugs were resolved by a software developer immediately. Following prioritization by the project team, high-priority change requests were implemented using the same software development protocol (eg, QA, UAT).

### Beta-Phase Testing

The aim of beta-phase testing was to prepare for the public release of AOPOC. It included load testing to quantify and improve system performance, usability testing to again evaluate ease of learning, and using an updated version of AOPOC.

#### Load Testing

To determine AOPOC performance capabilities, a test harness was developed that ran scripts which simulated common use cases identified by the business analyst and informatics project manager. With this test harness, load testing was conducted by generating a simulated load for a typical AOPOC use case of registering a patient and administration of the default PRO assessment battery. The load testing gradually increased the number of simultaneous patients, starting with 100 and increasing until the system started to return timeout errors caused by server requests exceeding the defined default response time (90 sec). Rounds of testing were conducted to identify server settings that maximized performance, identified bottlenecks (area within the software application that slowed overall performance because of concurrency), and reevaluated performance after modifications were implemented. A benchmark to double-simulated patients was established.

#### Usability

Surgeon members of AO Trauma, a nonprofit international organization of clinicians and researchers aimed at fostering and improving medical care for musculoskeletal trauma, were surveyed about their interest in serving as an AOPOC beta-test site. The project team reviewed 66 interested orthopedic clinics to identify representatives from specific types of clinics (eg, academic medical center, community hospital practice) and invited a diverse group of 36 sites to participate with a goal of enrolling 20. After 6 months, 16 sites had completed enrollment. Similar to alpha testing, usability feedback was collected in multiple ways. First, data were extracted from AOPOC to evaluate clinicians’ ability to tailor assessment content and patient response burden with PROMIS CATs. Second, interactions with clinical users including emails and calls to the AOPOC support desk and questions, comments, or difficulties during demonstrations were used to identify bugs as well as areas of confusion and errors. Third, beta-site leaders identified active AOPOC users at their sites to participate in a 20-min semistructured interview by phone. Interviews utilized the same modular guide as alpha testing. All issues, requested modifications, and responses to multiple-choice items were recorded in a database. Again, bugs were identified throughout beta testing and resolved immediately. Following prioritization by the project team, all high-priority change requests that fit within the available resources were implemented using the same software development protocol (eg, QA, UAT).

### Analytic Plan

#### Alpha-Phase Patient-Level Data

All patient-level data were exported from AOPOC by a software developer and deidentified by a data manager following a standardized protocol. To evaluate measurement performance, score distributions and mean PROMIS scores were calculated for each PROMIS CAT. Completion time was evaluated with frequency distributions for the number of items administered and time to complete each CAT. Frequency distributions and means for individual usability survey items were calculated.

#### Alpha-Phase Clinician-Level Data

The database of user feedback was reviewed, and redundant entries combined noting the number of users reporting the same issue. If required, additional clarification was sought from the source. All change requests were prioritized for implementation using a Must, Should, Could, or Would rating. Each change request was categorized as a Must (a mandatory change for the intended functionality of AOPOC), Should (a high-priority and desirable change that is not mandatory), Could (a change that would improve AOPOC but is not critical to its success), or Would (a change that may be considered but is not critical or appropriate now) rating. Ratings were based upon (1) alignment with the intended scope of AOPOC, (2) number of affected users, (3) frequency of request, and (4) whether a user could circumvent the issue. The project leader made an initial rating assignment. This was provided to the project team who reviewed and revised ratings individually and then met for a consensus meeting to finalize ratings. The available development effort was used to implement change requests in order of priority until it was expended.

#### Beta-Phase Usability Data

All beta-phase patient-level data were exported by a software developer and deidentified by a data manager following a standardized protocol. All available data were included in analysis, including sites which did not participate in usability interviews. Descriptive statistics including the number of Patient Groups set up within each beta test site, number of measures in addition to the AOPOC battery of measures that were administered per Patient Group, and number of unique patients providing data were calculated. Frequency distributions were constructed for the use of other PROMIS CATs and number of items needed to complete the default and supplemental PROMIS CATs.

*Should* change requests that were identified but not implemented during alpha testing were merged with issues identified in beta testing. All were reviewed, and redundant entries were combined. Potential system modifications to address user issues were identified. All change requests were prioritized using new Must, Should, Could, or Would ratings. The project leader provided the initial prioritization, distributed it to the project team, and a meeting was held to reach consensus on ratings. The available development effort was used to implement change requests in order of priority until it was expended.

## Results

### Alpha-Phase Testing

#### Patients

Across the three sites, 1793 unique patients were registered in AOPOC and eligible to complete 2640 assessments across all office visits. About half (935/1793, 52.14%) completed an initial assessment. Of the 614 eligible, 160 (26.1%) completed a second assessment at a follow-up clinic visit. Across all office visits, almost all of the registered patients who did not complete an assessment (1505/1511, 99.60%) were from one site that registered patients the day before his or her clinic visit.

##### Patient-Reported Outcome Scores

**Figure 6:**
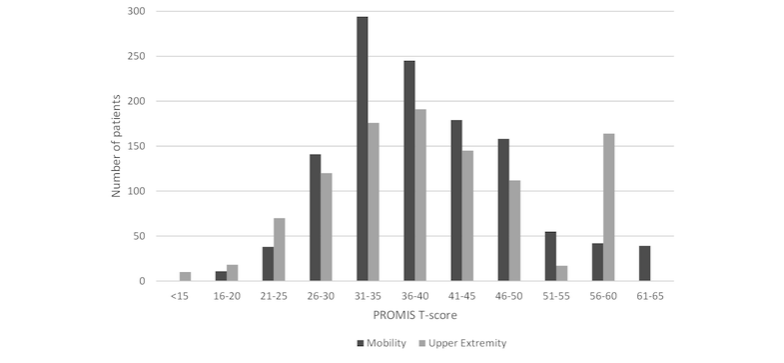
Frequency distribution of Patient-Reported Outcomes Measurement Information System (PROMIS) mobility and upper extremity function computer adaptive test (CAT) T-scores.

**Figure 7:**
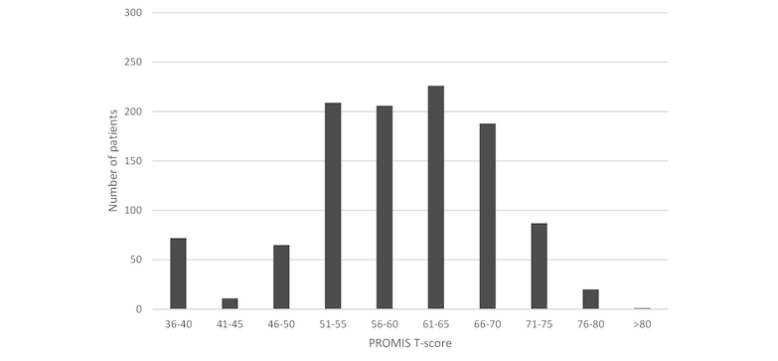
Frequency distribution of Patient-Reported Outcomes Measurement Information System (PROMIS) Pain Interference computer adaptive test (CAT) T-scores.

In alpha testing, separate Mobility and Upper Extremity Function CATs were used instead of a single Physical Function CAT. Half of the subjects completed the entire assessment battery (3 CATs) in under 3.7 min (mean 4.7 [SD 5.2], 95% CI 4.5-5.0). Long completion times were partly because of interruptions from the clinic staff (eg, moving the patient to the visit room). About 85% of patients answered only the minimum of 4 questions for the Mobility (781/923, 84.6%) and Pain Interference (766/897, 85.4%) CATs compared with only 22.1% (200/907) of patients for the Upper Extremity CAT. For the Upper Extremity CAT, 42.3% (384/907) of patients answered the maximum number of questions which was 12 (Table 1). Mean T-scores were in the moderate impairment range for Mobility (mean 38.7 [SD 8.7], 95% CI 38.1-39.2) and at the border among mild symptoms and within normal limits for Pain Interference (mean 59.2 [SD 9.4], 95% CI 58.6-59.9) with fairly well-distributed scores (Figures 6 and 7). Upper Extremity Function was also in the moderate impairment range (mean 39.8 [SD 10.7], 95% CI 39.1-40.5). The best possible score (T=56.4) was received by 15.7% (142/907) of patients, indicating that the measure may be unable to distinguish among patients with excellent function (*ceiling effect*).

**Table 1:** Performance of instruments in the AO Patient Outcomes Center battery.

Measure	Completion time (seconds)		Number of items administered		Assessments with minimum number of items, n (%)	Assessments with maximum number of items, n (%)	T-score	
	Mean (SD)	Median (min-max)	Mean (SD)	Median (min-max)			Mean (SD)	Median (min-max)
PROMIS^a^ Mobility CAT^b^ (n=923)	131.5 (133.7)	85.0 (5.0-1108.0)	4.7 (2.1)	4.0 (4-12)	781 (84.6)	69 (7.5)	38.7 (8.7)	37.2 (18.3-60.2)
PROMIS Upper Extremity CAT (n=907)	96.6 (109.3)	70.0 (4.0-1010.0)	8.2 (3.5)	7.0 (4-12)	200 (22.1)	384 (42.3)	39.8 (10.7)	38.7 (14.7-56.4)
PROMIS Pain Interference CAT (n=897)	60.0 (69.7)	42.0 (4.0-790.2)	4.8 (2.3)	4.0 (4-12)	766 (85.4)	76 (8.5)	59.2 (9.4)	60.1 (38.5-80.1)

^a^PROMIS: Patient-Reported Outcomes Measurement Information System.

^b^CAT: computer adaptive test.

##### Usability

Overall, most patients were familiar with using computers; 85.9% (736/857) used a computer within the past year. A similar number (724/856, 84.6%) used a touchscreen such as automated teller machine or airline check-in kiosk. A majority (721/839, 85.9%) owned a device with internet connectivity and reported use 5 to 7 days a week (551/741, 74.4%). AOPOC was not difficult for patients to use (Table 2). Most participants (686/842, 81.5%) reported they had no difficulty using the data collection device (tablet or desktop computer). Only 7% (58/840, 6.9%) were “somewhat” or “quite a bit” uncomfortable, anxious, or nervous using the data collection device. Most (721/841, 85.7%) had no difficulty answering the PRO questions. A strong majority (760/839, 90.6%) would be willing to complete a similar assessment at a future clinic visit. Furthermore, ratings of the AOPOC interface design were favorable, including data collection screens (741/843, 87.9% good, very good, or excellent) and the response button design (743/825, 90.1% good, very good, or excellent; Table 3).

**Table 2:** Usability of AO Patient Outcomes Center for patients.

Questions	Not at all, n (%)	A little bit, n (%)	Somewhat, n (%)	Quite a bit, n (%)
Did you have any difficulty using this computer? (N=842)	686 (81.5)	89 (10.6)	35 (4.2)	32 (3.8)
Did you ever feel uncomfortable, anxious, or nervous while using the computer? (N=840)	685 (81.5)	97 (11.5)	33 (3.9)	25 (3.0)
How difficult was it to answer the questions shown on this computer? (N=841)	721 (85.7)	77 (9.2)	27 (3.2)	16 (1.9)

**Table 3:** Patients’ satisfaction with design of AO Patient Outcomes Center.

Questions	Excellent, n (%)	Very good, n (%)	Good, n (%)	Fair, n (%)	Poor, n (%)
What is your overall rating of the design of the screens including the colors and layout? (N=843)	182 (21.6)	256 (30.4)	303 (35.9)	87 (10.3)	15 (1.8)
What is your overall rating of the buttons on the screens, including their size and shape? (N=825)	197 (23.9)	275 (33.3)	271 (32.8)	65 (8.0)	17 (2.1)

#### Clinicians

Of the 13 clinic staff who were invited, 11 participated in a usability interview. Interviewees were from all 3 sites and were providers (surgeon, physician assistant; n=5), research staff (n=3), or in other positions (nurse manager, program director, medical secretary; n=3). One provider did not personally interact with AOPOC and therefore questions concerning application usability were skipped. All 3 sites utilized a single Patient Group with only the default assessment content. Each site followed a slightly different workflow (eg, staff register patient in AOPOC when he or she checks in, staff registers all patients for the following day). Data collection occurred during an existing wait time, such as in the clinic waiting room, on an iPad or in the visit room on an iPad or desktop computer.

Clinicians described feeling comfortable using AOPOC at the time of the interview (mean 4.7 [SD 0.5], n=10). AOPOC was easy to learn (mean 4.7 [SD 0.7], n=10) and easy to use (mean 4.5 [SD 0.8], n=10). The process for completing the application for access to AOPOC was described as “A little bit” difficult by the 2 respondents who did this task. Registering as a clinical user was “Not at all” or “A little bit” difficult for the 2 clinicians who answered this question. Clinicians who set up a Patient Group reported it as not difficult (mean 1.3 [SD 0.5], n=4). Clinicians also had little difficulty registering patients (mean 1.4 [SD 0.7], n=8), starting a patient’s assessment (mean 1.3 [SD 0.8], n=6), and accessing a single patient’s data (mean 1.8 [SD 0.8], n=5). Only 2 attempted to export data from a Patient Group and both reported no difficulty. Areas where clinicians experienced more difficulty included entering clinical data (eg, fracture classification code, mean 3.5 [SD 2.1], n=2) and understanding the patient’s data including PROMIS CAT scores (mean 3.0 [SD 1.3], n=6). There was a wider range in responses concerning how disruptive AOPOC was to the clinic workflow (mean 2.3 [SD 1.2], n=9).

The qualitative feedback from the usability interviews and queries to the help desk identified 17 bugs and 104 change requests. Of the 17, 10 bugs were resolved during the pilot phase. The remaining bugs could not be recreated (n=4), they were recategorized to change requests (n=2) or required additional investigation time after the alpha-test period (n=1). Change requests were clustered in several areas. First, the process to register as a new site, register as a clinical user, and to provide access to other site members was reported to be challenging. Second, there was technical difficulty in utilizing an iPad as a data collection device. For example, users were not aware of the setup requirements to enable AOPOC on an iPad (eg, turn off pop-up blocker). Multiple clinicians commented that the staff had more difficulty interacting with an iPad than patients. Although support material including instructions for iPad setup was available, some users did not know it existed. Finally, the AOPOC Administrator identified multiple areas of difficulty in registering new users, including being unable to view those who were sent a registration email but had not completed the registration process, restrictions in the ability to make edits to user email addresses, health care organization names, and demographic fields (eg, State). In the consensus meeting, 17 *Must*, 31 *Should*, 44 *Could*, and 12 *Would* change requests were identified. The Must requests were implemented before the beginning of beta testing. In addition, on the basis of the range restriction of the PROMIS Mobility and Upper Extremity Function CATs, these measures were replaced with the PROMIS v1.2 Physical Function CAT.

### Beta-Phase Testing

#### Load Testing

Throughout beta testing, no users reported problems with system speed. However, load testing identified 2 Web server settings that contributed to reduced speed: *keep alive* and the maximum number of connections. Tests using simultaneous requests and an initiation rate of 2 to 4 sec between requests were conducted. Changing default Microsoft Internet Information Services 7 settings and increasing the number of central processing units (CPUs) did not increase the number of requests. After suspecting that requests were being blocked from finishing, the extra CPU used on the staging database was applied to the staging Web server that was reaching capacity. This resulted in doubling the performance of requests finishing in multiple rounds of testing. Several recommendations were identified to prepare for wider distribution of AOPOC, including (1) updating hardware and increasing CPUs to 4, (2) continuous monitoring of performance by server host, and (3) engaging in ongoing evaluation with updated information about volume of simultaneous users.

#### Usability

Of the 16 enrolled sites, 8 had completed business associate agreements (BAAs) between their institution and AOPOC at the time of usability interviews. The remaining 8 sites completed BAAs after usability interviews were concluded, but they were able to provide data on the initial setup of AOPOC at their clinics. Reasons for not enrolling as a test site included no support from other clinic personnel, utilization of other data collection systems (eg, state-mandated system), and difficulties in executing a BAA. BAA challenges included (1) difficulty in identifying who had the authority to sign this agreement, (2) institutional staff requiring modifications to the existing BAA, which required negotiation (eg, removing AO Foundation’s right to use a deidentified dataset from AOPOC for research purposes), and (3) requiring that the local institution’s own standard BAA be utilized, requiring review and discussion with the project team.

Across 16 sites, 71 unique Patient Groups were created. Sites utilized 1 of 3 approaches to organizing Patient Groups: (1) a single Patient Group for all patients being treated by the same surgeon, (2) 2 large groups based upon location of injury (ie, upper extremity and lower extremity), or (3) multiple Patient Groups based upon location of injury (eg, acetabulum, elbow, foot or ankle, forearm or wrist, hip or femur, knee or tibia, pelvis, polytrauma, and shoulder or humerus). There was a wide range (2 to 24) of patient- and clinician-completed instruments per Patient Group. Approximately 34% (24/71) only collected the default battery. Between 3 and 5 additional instruments were included in 38% (27/71) of Patient Groups. Added measures were other PROs (47%, 153/326) and injury or treatment information (eg, mechanism of injury; 48%, 155/326). There were 9725 assessments completed by 5088 patients. Most patients completed 1 or 2 assessments, and the maximum completed by the same person was 14. The default assessment (2 CATs) required 9.5 items on average. For Pain Interference, 80% (6355/7895) of the assessments required only the minimum number of items per CAT (see Table 4). Similarly, 78% (6178/7965) of Physical Function assessments used the minimum number of items. An additional 8 PROMIS CATs were administered by at least one clinician. Most (n=6) were completed with 4 to 5 items. Depression (mean 7.2 [SD 3.8]) and Upper Extremity Function (mean 8.0 [SD 3.5]) required the most items, but in both cases, the number of items was bimodally distributed, with a substantial number completing the CAT in 4 or 5 items (for Depression and Upper Extremity Function, respectively) and a minority requiring the maximum of 12 items.

**Table 4:** Utilization and average length of Patient-Reported Outcomes Measurement Information System (PROMIS) computer adaptive tests (CATs) in beta phase.

PROMIS CAT	Assessments^a^	CAT length (items), mean (SD)	Assessments with minimum items, n (%)	Assessments with maximum items, n (%)
Pain interference (default assessment)	7895	5.0 (2.5)	6355 (80.49)	857 (10.85)
Physical function (default assessment)	7965	4.5 (1.4)	6178 (77.56)	167 (2.10)
Upper extremity function	488	8.0 (3.5)	118 (24.2)	191 (39.1)
Mobility	636	5.8 (3.2)	452 (71.1)	132 (20.8)
Pain behavior	78	4.7 (2.2)	70 (89.7)	6 (8)
Depression	39	7.2 (3.8)	20 (51)	14 (36)
Anxiety	19	4.9 (2.5)	17 (89)	2 (11)
Fatigue	75	4.3 (1.2)	67 (89)	1 (1)
Sleep disturbance	37	5.1 (2.4)	19 (51)	2 (5)
Ability to participate in social roles and activities	18	4.7 (1.9)	14 (78)	1 (6)

^a^Some patients completed more than 1 assessment.

A total of 16 clinicians were invited to participate in a usability interview. Of these, 10 were not yet using AOPOC in clinic. A total of 5 clinicians across 4 sites completed the interview. All interview participants reiterated the ease of learning and using AOPOC. The most significant concern was the impact of PRO collection on clinic workflow. For example, patients completing an assessment in the exam room did not always finish the assessment before the surgeon arrived, making PRO information unavailable in the clinic visit. Aligning the assessment with existing wait time (eg, waiting for x-ray, waiting for exam room) required trial and error and was not always consistent across patients. Some clinicians carried a tablet computer to view scores, which was additional equipment with a new log-in. Adding a 1-min task for the staff was significant when a clinic included more than 40 appointments. Change requests were related to usability (eg, alphabetize patient list, use consistent messaging with *save* buttons, ensure graphical report fits on a standard paper size, and orient all graphs so that higher points are consistently good), security (eg, institution-specific tablet computer settings), and default assessment content (eg, appears to be a ceiling of possible high scores, weight-bearing activity limitations should be in default assessment, and minimal questions on Upper Extremity Function). Following prioritization, 9 change requests related to usability were evaluated as *Musts* and implemented (eg, improve consistency in use of checkboxes, increase visibility of location within the application, and clarify reminders to clinicians about missing clinical information). Of these, 2 were also reported and rated as *Shoulds* during the alpha-phase testing. A total of 6 bugs were identified with only 1 being critical—1 test site blocked incoming registration emails from AOPOC. All bugs were resolved during the beta-phase testing. In addition, 9 security improvements (eg, server-side validation, improved encryption of URLs, construction of log of all user log-ins and failed log-ins) were implemented.

## Discussion

### Summary

AOPOC was demonstrated to be an easy-to-learn and easy-to-use software application for patients and clinicians that can be integrated into routine orthopedic care. In alpha-phase testing, a battery of PROMIS CATs was completed in under 5 min with usually only the minimum number of questions required. The measures performed well, although Upper Extremity Function demonstrated a limitation in assessing patients with better functioning. Patients were comfortable using the data collection device and answering questions with 90.6% (760/839) willing to complete an assessment again. Clinicians also reported AOPOC was easy to learn and its multiple features were easy to use. The most frequently cited areas for improvement were in onboarding as a new site and clinical user and in mitigating the impact of PRO collection on the clinic workflow. A small number of bugs were reported, none of which were critical (ie, prevented data collection). In beta-phase testing, areas for improving system speed were identified and addressed. PROMIS CATs performed well; they were completed quickly and there were no ceiling effects in the revised default assessment. Only 1 critical bug was identified, which was resolved. Clinicians demonstrated the ability to establish more complex assessment plans through multiple assessment types and tailored content. Of the small number of clinicians who completed interviews, concerns included executing a BAA and achievable future modifications to improve usability.

### Patient-Reported Outcomes in Orthopedic Clinical Practice

AOPOC addresses some of the barriers to the collection and use of PROs in routine orthopedic clinical practice. First, similar to other studies in orthopedic patient populations [13,14], the PROMIS Physical Function, Mobility, and Pain Interference CATs demonstrated acceptable assessment at the very high and very low ends of the range. However, the PROMIS v1.2 Upper Extremity Function CAT did demonstrate poor ability to distinguish among levels of excellent function and therefore may not be able to capture the full degree of improvement following intervention. CATs also remove a second assessment barrier—time burden [15]. By tailoring the items that are administered, measures were most frequently completed in 4 to 5 items. Finally, AOPOC provided measure scores immediately in table and graphical presentations, thus removing the need to calculate and display results [16].

This project highlights 2 issues related to integrating PRO collection in clinical practice that are not specific to AOPOC: (1) managing the impact of adding a PRO assessment to the clinic workflow is critical and (2) the site-specific requirements for utilizing a software application in parallel with an EHR are substantial. In this project, clinic workflow disruption was frequently identified as a concern, which is consistent with previous research [5]. Low participation from clinicians at beta-test sites and by registered patients at 1 alpha-test site may also reflect workflow problems. Collection of PROs requires patients’ time to complete measures and care providers’ time to access and review results [17]. Enabling patients to complete assessments outside of the clinic setting can reduce the time demand on patients at the care setting, though participation has been low [18,19]. Another strategy has been to improve the usefulness of PRO results to increase patients’ and clinicians’ engagement in PRO collection. Aligning the assessment content with the clinical purpose (eg, screening vs monitoring a primary outcome of care) [18,20] and making PRO results more interpretable and actionable [21-24] have been recommended. Clinician training programs have also been successful in improving interpretation and the use of PRO results [25].

Software apps that work in parallel with an EHR require BAAs. Most of the beta-testing sites required more than 4 months for a BAA to be finalized and some sites were not successful in reaching an agreement even after 8 months. As reimbursement is increasingly tied to PRO collection [7,26], the prioritization by health care systems to enable PROs in clinical care is expected to increase. Non-EHR data collection software apps like AOPOC are able to quickly integrate advances in PRO measurement science such as improved measures (eg, a PROMIS Upper Extremity CAT with a higher measurement ceiling) and graphical displays of results (eg, integrate newly published normative scores for a particular patient population). Without increasing the efficiency of adopting software apps into clinical care, the breadth of advances in patient-centered care will remain difficult to implement.

### Limitations

Several limitations are noted. Most of the patient participants had previous experience interacting with a computer and a touch screen. Consequently, the positive usability feedback may not represent the experience of those patients without past experience. Information on the number of patients who were approached to complete an assessment but declined was not captured. Therefore, it is not known to what extent patient factors (eg, computer fluency, concerns about privacy, and English literacy) and/or clinic factors (eg, barriers to adding an assessment to clinic workflow, insufficient data collection devices) contributed to patients not completing an assessment. Similarly, there may have been selection bias inherent in getting clinician feedback from those who used the system; clinicians who were more comfortable or open to computerized testing might have been more likely to use AOPOC. In addition, both alpha and beta testing occurred in the clinics of orthopedic surgeons who were members of the AO Foundation, which may not be representative of all orthopedic surgeons.

### Conclusions

AOPOC is a feasible, robust software application that enables collection of CATs within orthopedic clinical care. Barriers to routinely integrating PROs, including modification of clinic workflow and execution of BAAs remain. However, addressing those barriers enables the integration of the patient’s perspective in his or her health care. This is particularly important in orthopedics as physical function and pain are regularly the targets of interventions and the reasons why patients seek care.

## Abbreviations

AOPOC: AO Patient Outcomes Center
BAA: business associate agreement
CAT: computer adaptive test
CPU: central processing unit
EHR: electronic health record
PRO: patient-reported outcome
PROMIS: Patient-Reported Outcomes Measurement Information System
QA: quality assurance
UAT: user acceptance testing
